# Supplementary material for: Effects of plastic mulching on the accumulation and distribution of macro and micro plastics in soils of two farming systems in Northwest China
Source: PeerJ. 2020 Dec 1;8:e10375. doi: 10.7717/peerj.10375 (PMC7718786; doi:10.7717/peerj.10375)
Supplement: Supplemental Information 1 [file peerj-08-10375-s001.docx]

**Supplementary file of “Effect of plastic mulching on the accumulation and distribution of macro and micro plastics in soils of two farming systems in Northwest China”**

Fanrong Meng, Tinglu Fan, Xiaomei Yang, Michel Riksen, Minggang Xu, Violette Geissen

**Table S1:** Climate data and soil information

**Table S2:** Plastic specification used in S1 and S2

**Table S3:** Summary of the one way ANOVA for macroplastics number and content in 0-30 cm.

**Table S4:** The number and mass percentage of MaPs in different size categories in two study region

**Table S5:** Summary of the one way ANOVA for the number and mass percentage of MaPs in different size categories in two study region

**Table S6:** Summary of the one way ANOVA for macroplastics number and mass in different soil layer in S1

**Table S7:** Summary of the one way ANOVA for macroplastics number and mass in different soil layer in S2

**Figure S1:** PP flat weave mesh bag.

**Figure S2:** Macroplastics shapes.

**Figure S3:** Macroplastics size group classification.

**Figure S4:** PP bag for collecting soil samples for microplastic extraction.

**Figure S5:** Microplastics identification set up.

| **Table S1:** Climate data and soil information. | | |
| --- | --- | --- |
| Indicators | S1 | S2 |
| Annual average temperature | 7-10°C | 6.5-7.2 °C |
| Accumulate temperature (>10℃) | 2 722 ℃ | 2 950 ℃ |
| Annual average precipitation | 540 mm | 210 mm |
| Annual sunshine duration | 2 262 h | 2 865 h |
| Frost-free days | 160 days | 160 days |
| Annual average wind speed | 2.16 m/s | 1.75 m/s |
| Soil type | Calcarid Regosol | Calcaric Fluvisol |
| Soil pH | 7.98±0.14 | 7.90±0.05 |
| Soil organic matter content (SOM) | 13.1±1.76 g·kg^-1^ | 14.3±0.44 g·kg^-1^ |
| **Note.** SOM measured by method:H_2_SO_4_-K_2_Cr_2_O_7_.Soil pH and soil organic matter (SOM) content was the average of selected fields in each study region. | | |

| **Table S2:** Plastic specification used in S1 and S2 | | | | | | |
| --- | --- | --- | --- | --- | --- | --- |
| Plastic film | Colour | Thickness (mm) | Density (g·cm^−3^) | Materials | Standard | Manufacturer |
| S1 | Transparent | 0.01 | 0.9 | LLDPE | GB 13735-1992(II) | Gansu Swift Management Consulting Co., Ltd., China |
| S2 | Transparent | 0.01 | 0.9 | LLDPE & LDPE | GB 13735-1992(IV) | Xinjiang Tianshili Plastic Industry Co., Ltd. |
| **Note:** low-density polyethylene (LDPE) and linear low-density polyethylene (LLDPE). | | | | | | |

| **Table S3:** Summary of the one way ANOVA for macroplastics number and content in 0-30 cm. | | | | |
| --- | --- | --- | --- | --- |
| MaPS accumulation | Study region | df | F | Sig |
| MaPs number | S1 | 5,12 | 20.9 | 0.00 |
|  | S2 | 4,10 | 61.7 | 0.00 |
|  |  |  |  |  |
| MaPs mass | S1 | 5,12 | 4.24 | 0.02 |
|  | S2 | 4,10 | 17.1 | 0.00 |

| **Table S4 :** The number and mass percentage of MaPs in different size categories in two study region (one way ANOVA and followed by LSD test at the p <0.05 level). | | | | | | | | | | | |
| --- | --- | --- | --- | --- | --- | --- | --- | --- | --- | --- | --- |
| MaPs accumulation | Fields | 0.25-2 cm² | | 2-10 cm² | 10-50 cm² | | 50-100 cm² | | >100 cm² | |  |
| MaPs number | S1 Continuous | 3.55±2.16d | | 34.4±8.19b | 46.9±7.64a | | 9.57±2.57c | | 5.55±2.05d | |  |
|  | S1 Intermittent | 4.20±3.21d | | 31.6±9.63b | 44.5±11.2a | | 8.73±4.77c | | 11.0±10.2c | |  |
|  | S2 | 40.6±13.0a | | 41.1±9.43a | 14.7±4.71b | | 2.54±2.04c | | 1.09±1.13c | |  |
|  |  |  | |  |  | |  | |  | |  |
| MaPs mass | S1 Continuous | 0.16±0.09d | | 7.17±2.23c | 35.8±5.27a | | 22.0±4.50b | | 34.8±8.24a | |  |
|  | S1 Intermittent | 0.26±0.31d | | 6.28±3.91c | 34.2±21.6a | | 16.3±7.29b | | 42.9±24.0a | |  |
|  | S2 | 8.93±5.19c | | 26.2±7.95b | 36.4±7.1a | | 17.4±8.06c | | 11.1±10.8c | |  |
| **Note:** Lowercase letters (a,b,c) indicate significant difference between different MaPs size categories within same fields. | | | | | | | | | | |  |
| **Table S5**: Summary of the one way ANOVA forthe number and mass percentage of MaPs in different size categories in two study region. Data have been transformed using arcsine square root. | | | | | | | | | | | |
| MaPs accumulation | | | Fields | | | df | | F | | Sig. | |
| MaPs number | | | S1 Continuous | | | 4,40 | | 148 | | 0.00 | |
|  |  |  | S1 Intermittent | | | 4,40 | | 35.9 | | 0.00 | |
|  |  |  | S2 | | | 4,70 | | 18.4 | | 0.00 | |
|  | | |  | | |  | |  | |  | |
| MaPs mass | | | S1 Continuous | | | 4,40 | | 217 | | 0.00 | |
|  |  |  | S1 Intermittent | | | 4,40 | | 28.4 | | 0.00 | |
|  |  |  | S2 | | | 4,70 | | 172 | | 0.00 | |

| **Table S6:** Summary of the one way ANOVA for macroplastics number and mass in different soil layer in S1. Data have been transformed using arcsine square root. | | | | |
| --- | --- | --- | --- | --- |
| MaPs accumulation | Study region | df | F | Sig |
| MaPs number | S1-1 | 2,6 | 12.1 | 0.01 |
|  | S1-2 | 2,6 | 16 | 0.00 |
|  | S1-3 | 2,6 | 39.4 | 0.00 |
|  | S1-4 | 2,6 | 7.74 | 0.02 |
|  | S1-5 | 2,6 | 11.7 | 0.01 |
|  | S1-6 | 2,6 | 4.88 | 0.06 |
|  |  |  |  |  |
| MaPs mass | S1-1 | 2,6 | 28.9 | 0.001 |
|  | S1-2 | 2,6 | 22.2 | 0.002 |
|  | S1-3 | 2,6 | 46.7 | 0.000 |
|  | S1-4 | 2,6 | 9.19 | 0.015 |
|  | S1-5 | 2,6 | 4.38 | 0.067 |
|  | S1-6 | 2,6 | 12.6 | 0.007 |

| **Table S7:** Summary of the one way ANOVA for macroplastics number and mass in different soil layer in S2. Data have been transformed using arcsine square root. | | | | |
| --- | --- | --- | --- | --- |
| MaPs accumulation | Study region | df | F | Sig |
| MaPs number | S2-1 | 2,6 | 4.51 | 0.064 |
|  | S2-2 | 2,6 | 4.83 | 0.056 |
|  | S2-3 | 2,6 | 4.5 | 0.064 |
|  | S2-4 | 2,6 | 32.5 | 0.001 |
|  | S2-5 | 2,6 | 6.79 | 0.029 |
|  |  |  |  |  |
| MaPs mass | S2-1 | 2,6 | 5.55 | 0.043 |
|  | S2-2 | 2,6 | 24.6 | 0.001 |
|  | S2-3 | 2,6 | 16.9 | 0.003 |
|  | S2-4 | 2,6 | 18.7 | 0.003 |
|  | S2-5 | 2,6 | 19.2 | 0.002 |


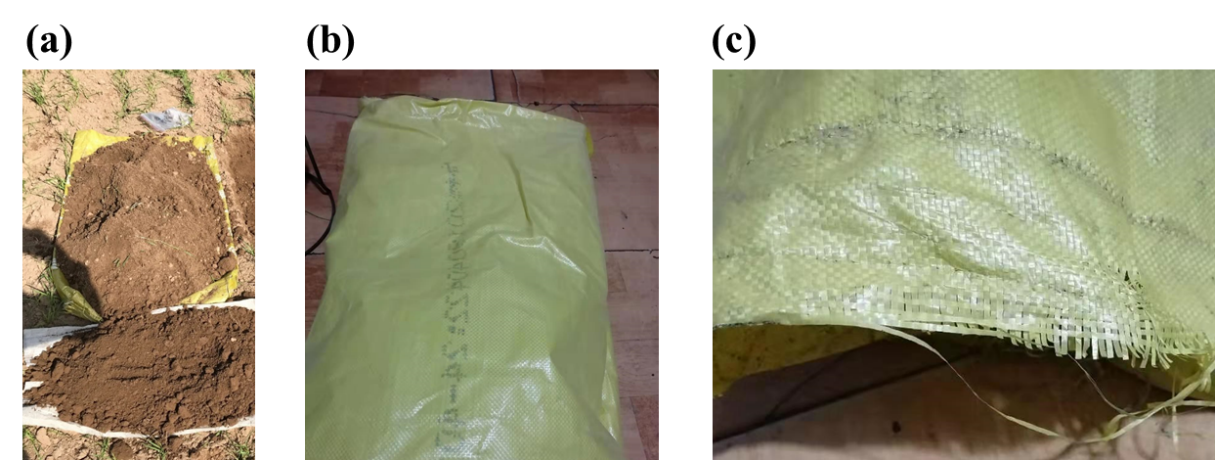


**Fig. S1.** Flat PP wire weaved mesh sheet. (a), (b) are the Flat PP wire weaved mesh sheet used in the field sampling, (c) is the flat wire used for weave the bag.


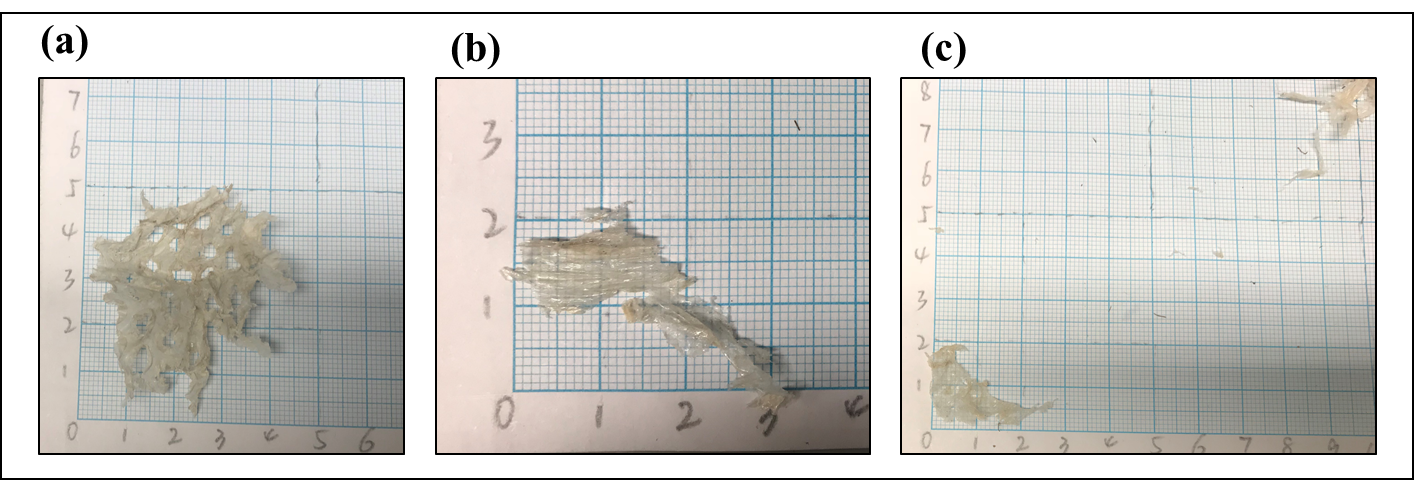


**Fig. S2.** The shapes and smallest size of collected Macroplastics. (a) arbitrary shape; (b) curved shape; (c) flake shape and curved shape; (d) the macroplastic buried in soil layers and collected macroplastics.


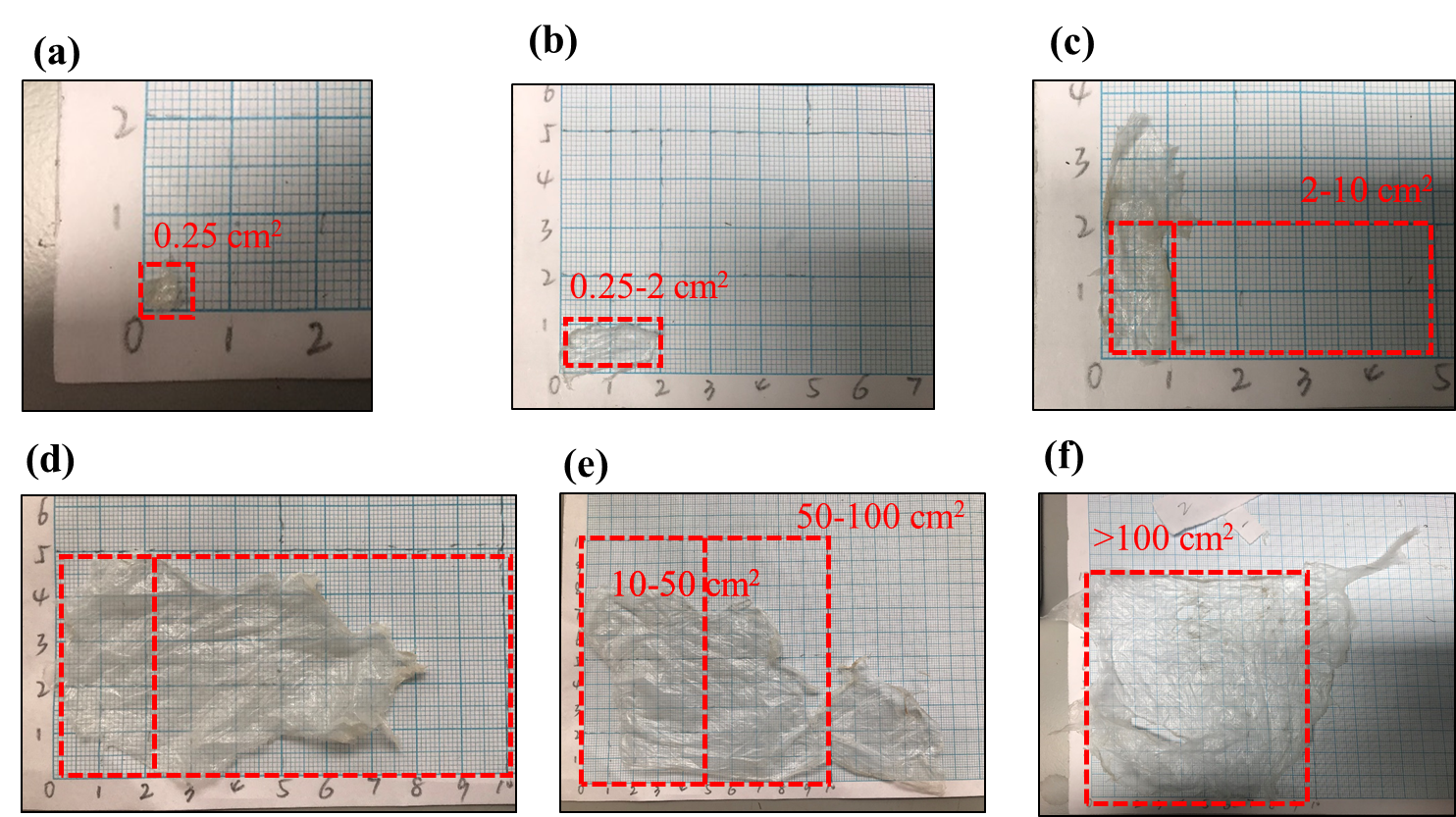


**Fig. S3.** Macroplastics different size group; (a) the smallest size of detected MaPs (approximately 0.25 cm^2^); (b) Macroplastics size 0.25-2cm^2^; (c) Macroplastics size 2-10 cm^2^; (d) Macroplastics size 10- 50 cm^2^; (e) Macroplastics size 50- 100 cm^2^; (f) Macroplastics size > 100 cm^2^.


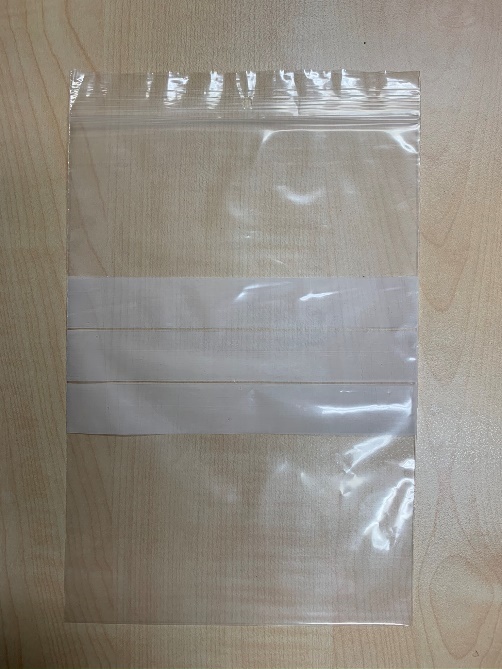


**Fig. S4.** PP bag for collecting soil samples for microplastic extraction.


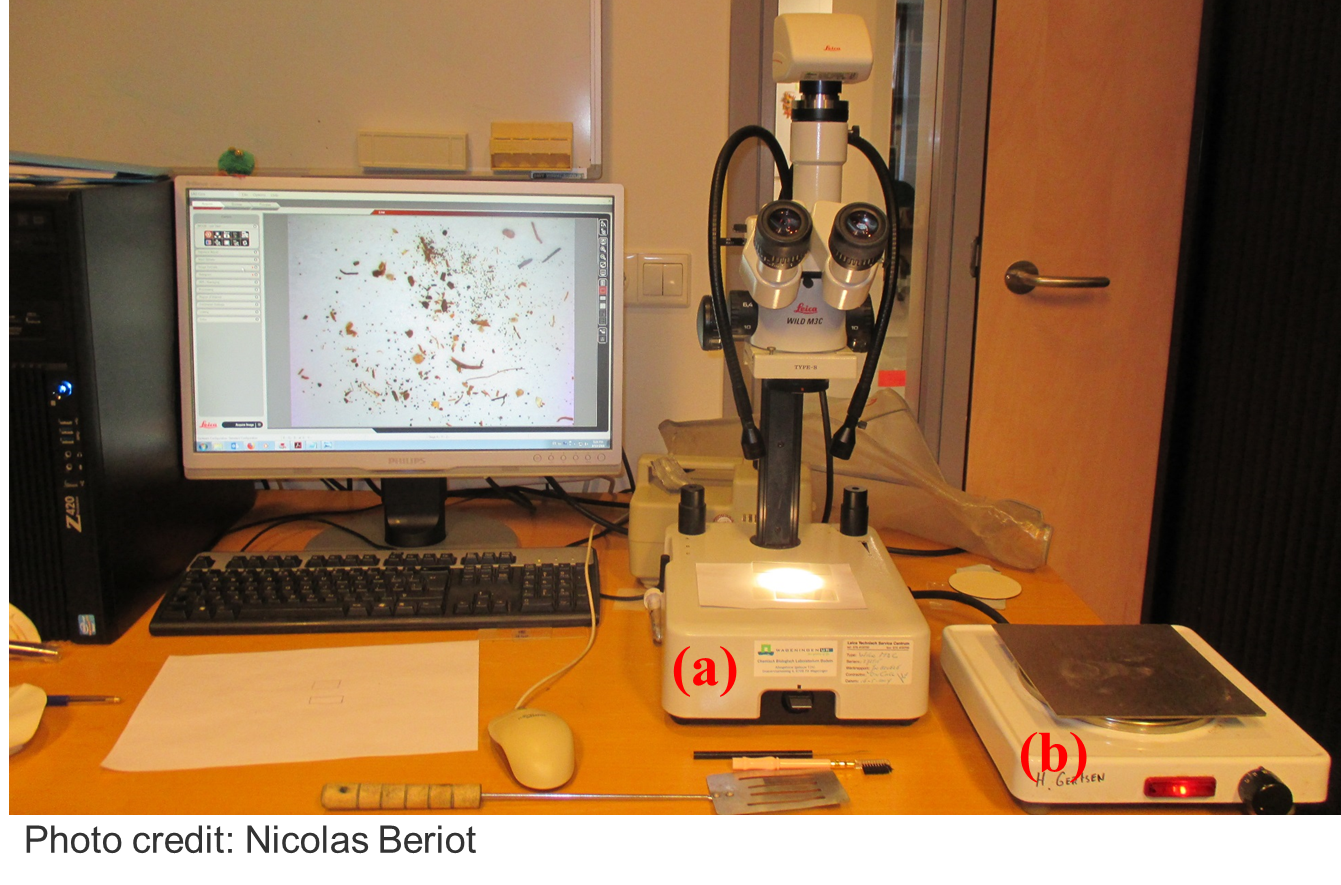


**Fig. S5.** Microplastics identification set up; (a) microscope, Leica wild M3C, Type S, simple light; (b) hot plate, TYPE A 1500-145, KEMA KEUR. ***Copyright:*** Beriot, Nicolas [nicolas.beriot@wur.nl](mailto:nicolas.beriot@wur.nl).
